# A Cannabinoid 2-Selective Agonist Inhibits Allogeneic Skin Graft Rejection *In Vivo*

**DOI:** 10.3389/fphar.2021.804950

**Published:** 2022-02-03

**Authors:** Senthil Jayarajan, Joseph J. Meissler, Martin W. Adler, Toby K. Eisenstein

**Affiliations:** Center for Substance Abuse Research, Lewis Katz School of Medicine at Temple University, Philadelphia, PA, United States

**Keywords:** graft rejection, CB2 agonist, T reg cells, mixed lymphocyte reaction (MLR), immunosuppresion

## Abstract

Previous work from our laboratory showed that a CB2 selective agonist, O-1966, blocked the proliferative response of C57BL/6 mouse spleen cells exposed to spleen cells of C3HeB/FeJ mice *in vitro* in the mixed lymphocyte reaction (MLR). The MLR is widely accepted as an *in vitro* correlate of *in vivo* grant rejection. Mechanisms of the immunosuppression induced by the cannabinoid were explored, and it was shown that O-1966 in this *in vitro* assay induced CD25^+^Foxp3^+^ Treg cells and IL-10, as well as down-regulated mRNA for CD40 and the nuclear form of the transcription factors NF-κB and NFAT in T-cells. The current studies tested the efficacy of O-1966 in prolonging skin grafts *in vivo*. Full thickness flank skin patches (1-cm^2^) from C3HeB/FeJ mice were grafted by suturing onto the back of C57BL/6 mice. O-1966 or vehicle was injected intraperitoneally into treated or control groups of animals beginning 1 h pre-op, and then every other day until 14 days post-op. Graft survival was scored based on necrosis and rejection. Treatment with 5 mg/kg of O-1966 prolonged mean graft survival time from 9 to 11 days. Spleens harvested from O-1966 treated mice were significantly smaller than those of vehicle control animals based on weight. Flow cytometry analysis of CD4^+^ spleen cells showed that O-1966 treated animals had almost a 3-fold increase in CD25^+^Foxp3^+^ Treg cells compared to controls. When dissociated spleen cells were placed in culture *ex vivo* and stimulated with C3HeB/FeJ cells in an MLR, the cells from the O-1966 treated mice were significantly suppressed in their proliferative response to the allogeneic cells. These results support CB2 selective agonists as a new class of compounds to prolong graft survival in transplant patients.

## 1 Introduction

The discovery of the CB2 receptor and its abundance and fairly selective expression on cells of the immune system (Munro et al., 1993; Galiegue et al., 1995) has posed the question of its function on immune responses. Many studies investigating beneficial or detrimental effects of cannabinoids on immune responses and resistance to infection have focused on Δ^9^-THC or on the endogenous cannabinoids, 2-arachidonoylglycerol (2-AG) and anandamide. All three of these ligands bind to both CB1 and CB2. To prove that an effect of these agonists occurs via the CB2 receptor, investigators have used selective CB1 and CB2 antagonists, or used CB1 or CB2 receptor knock-out mice. Another approach to probing the role of CB2 receptors in immune responses is to use synthetic, CB2 selective agonists (Huffman et al., 1996; Hanuš et al., 1999; Huffman et al., 1999; Pacher and Mechoulam, 2011). With these approaches, the majority of studies on cannabinoids have shown them to be anti-inflammatory, immunosuppressive (Croxford and Yamamura, 2005; Klein, 2005; Eisenstein and Meissler, 2015) and to polarize immune responses towards a Th2 phenotype (Newton et al., 1994; Klein et al., 2000; Yuan et al., 2002; Yang et al., 2014). Immunosuppression by Δ^9^-THC has been associated with increases in the immunosuppressive and anti-inflammatory cytokines, TGF-β and IL-10 (Zhu et al., 2000), and it was shown that TGF-β induction occurs via the CB2 receptor (Gardner et al., 2002). Δ^9^-THC has also been shown to inhibit macrophage presentation of antigen to T cells, which occurred through effects at the CB2 receptor, as macrophages taken from CB2 k/o mice were not suppressed (Buckley et al., 2000). Δ^9^-THC and anandamide suppressed *in vitro* antibody formation by mouse splenocytes in a CB2 dependent manner as determined using cannabinoid receptor selective inhibitors (Eisenstein et al., 2007). CB2 selective agonists have been shown to be broadly anti-inflammatory, inhibiting paw edema in a rat carrageenan model, which correlated with reduced neutrophil infiltration and decreased production of reactive oxygen intermediates (Parlar et al., 2018). CB2 agonist have also been reported to inhibit chemotaxis of primary human blood T cells and the human Jurkat cell line to the chemokine CXCL12 (Ghosh et al., 2006; Coopman et al., 2007). Antigen-specific and non-antigen specific T cell proliferation was inhibited by CB2 agonists (Maresz et al., 2007), and by anandamide, acting through the CB2 receptor (Cencioni et al., 2010). Further, CB2 selective agonists have been shown to ameliorate autoimmune reactions in a variety of mouse models that include experimental autoimmune encephalitis (EAE) (a model for multiple sclerosis) (Ni et al., 2004; Maresz et al., 2007), systemic sclerosis (Akhmetshina et al., 2009; Servettaz et al., 2010), autoimmune uveoretinitis (Xu et al., 2007), murine colitis and inflammatory bowel disease (Storr et al., 2009; Singh et al., 2012; Fichna et al., 2014; Leinwand et al., 2017).

A model explored by our laboratory has been to test the effects of CB2 selective agonists on the mixed lymphocyte reaction (MLR). The MLR is accepted as an *in vitro* correlate of *in vivo* graft rejection. Briefly, spleen cells from two histoincompatible mouse strains are placed in culture together. Cells from the stimulator strain are inhibited from dividing by treatment with mitomycin C. After 48 h of incubation the responder strain cells will proliferate, which can be quantitated using tritiated thymidine. Our results have shown that CB2 selective agonists strongly inhibit the MLR. In the *in vitro* cultures IL-2 is inhibited, IL-10 is augmented, and Treg cells are induced (Robinson et al., 2013; Robinson et al., 2015). The present studies examined the effect of injecting a CB2 selective agonist on *in vivo* immune responses, including skin graft rejection in mice, and cytokine and Treg levels in treated, as compared to, animals receiving vehicle. A CB2 agonist was shown to prolong skin graft rejection time and to induce IL-10 and Tregs in the mouse spleen.

## 2 Methods

### 2.1 Cannabinoid

O-1966, a CB2-selective agonist, was a generous gift from Anu Mahadevan (Organix, Woburn, MA). The affinity of O-1966 for CB1 and CB2 cannabinoid receptors was reported previously to be 5,055 ± 984 and 23 ± 2.1 nmol/L, respectively (Wiley et al., 2002). It was shown to stimulate ^35^S-GTPγS binding with an EC50 of 70 ± 14 nmol/L and an Emax of 74 ± 5 (percent of maximal stimulation produced by the full agonist CP 55,940) (Zhang et al., 2007).

### 2.2 Mice

Six week-old, specific pathogen-free C3HeB/FeJ and C57BL/6J female mice were purchased from Jackson Laboratories (Bar Harbor, Maine). Animals were housed in the central animal facility of Lewis Katz School of Medicine at Temple University which is AAALAC certified. Treatments were carried out following procedures approved by the University IACUC Committee.

### 2.3 Experimental Design

#### 2.3.1 Skin Graft Procedure

All surgeries were done under aseptic conditions. Animals were anesthetized with 1–2% isofluorane delivered via nose cone. To test the capacity of O-1966 treatment to inhibit rejection of a skin graft *in vivo*, 1 cm^2^ pieces of flank skin were harvested from donor C3HeB/FeJ mice. The flank of recipient C57BL/6J mice was prepared to receive the graft by removing the skin and superficial tissue to create a graft bed slightly larger than the piece to be transferred. The graft was transferred into the bed and sutured in place. The graft was bandaged for 7 days. This procedure followed a standard protocol for carrying out such grafts (Lagodzinski et al., 1990). Animals did not receive post-operative analgesic as per permission from the IACUC, because of concerns that the analgesic might affect immune status which was being monitored as a function of cannabinoid treatment. Doses of O-1966 or vehicle (0.03% ethanol and 0.03% cremophor in saline) were administered by intraperitoneal injection (i.p.) every other day from 1 h before transplantation to post-operative day 14. Three different doses of O-1966 were used in three different cohorts of mice: 1, 5, and 10 μg/kg. Bandages were removed on day 7 and the grafts were monitored daily for rejection. An allograft was considered fully rejected when it was >90% necrotic. In the initial experiments, the three different dosage groups had 8 animals each. The experiment was repeated using just the 5 mg/kg dose, with 9 mice in the cannabinoid group and 9 in the vehicle group, yielding a total of 17 animals in each group when the two experiments at 5 mg/kg were combined. All animals were sacrificed at 14 days post-surgery in order to harvest splenic tissue to assess immune status as described below in Sections 2.3.2 and 2.3.3.

#### 2.3.2 One-Way Mixed Lymphocyte Reaction

C57BL/6J mice that had received skin grafts from C3HeB/FeJ mice, with or without treatment with O1966, were sacrificed 14 days after grafting surgery. Their spleens were aseptically removed, and single cell suspensions were obtained by passing spleens through nylon mesh bags (Sefar Inc., Depew, NY) in RPMI-1640 with 5% fetal bovine serum (FBS) containing 50 μM 2-mercaptoethanol (2-Me), and 100 U/ml penicillin and streptomycin sulfate. All reagents were purchased from Gibco Life Technologies (Carlsbad, CA), with the exception of FBS, which was purchased from HyClone Laboratories (Logan, UT). Red blood cells were lysed by hypotonic shock for 10 s with sterile water. Responder spleen cells from C57BL/6 mice were resuspended in RPMI with 10% FBS, 50 μM 2-Me, and 100 U/ml penicillin and streptomycin sulfate. Splenocytes from C3HeB/FeJ were similarly prepared to serve as the *in vitro* stimulator cells, but they were inactivated by treatment with 50 μg/ml of mitomycin C for 20 min at 37°C. The cells were washed three times to remove mitomycin C from the medium and resuspended to the desired concentration using a Beckman Coulter Z1 Dual Cell and Particle Counter (Beckman Coulter Inc., Indianapolis, IN). Responder cells (8 × 10^5^) and stimulator cells (8 × 10^5^) were co-cultured in 200 μl in 96 well plates for 48 h at 37°C in 5% CO_2_. After a 48 h incubation period, half of the cultures were tested to see if they responded to the stimulator cells by cell division in the MLR. The other half of the cells were analyzed by flow cytometry to determine the percentage of CD25^+^Foxp3^+^ Treg cells. To assay the MLR, cells were pulsed with 1 μCi/well [^3^H]-thymidine and harvested 18 h later onto glass fiber filters (Packard, Downers Grove, IL) using a Packard multichannel harvester, and placed in vials in liquid scintillation solution (Cytoscint, MP-Biomedical, Irvine, CA). [^3^H]-thymidine incorporation on the filters was measured using a Packard 1900 TR liquid scintillation counter. Data were corrected for background by subtraction of [^3^H]-thymidine incorporation in the absence of stimulator cells. Results are expressed as a suppression index (SI), where untreated spleen cells are given a value of 1.00 (100%), and responses of cultures receiving treatment with cannabinoids are calculated as:

$$\mathrm{SI}\;=\;\frac{\mathrm{Mean}\;\mathrm{counts}\;\mathrm{per}\;\mathrm{minute}\;\mathrm{of}\;\mathrm{cannabinoid}\;\mathrm{treated}\;\mathrm{cultures}}{\mathrm{Mean}\;\mathrm{counts}\;\mathrm{per}\;\mathrm{minute}\;\mathrm{of}\;\mathrm{untreated}\;\mathrm{cultures}}$$

The method for assaying for Treg cells is described below under the section on flow cytometry.

#### 2.3.3 Flow Cytometry

The MLR cultures were harvested at various time points and washed with staining buffer (PBS containing 1% BSA, Sigma, St. Louis, MO). 1 × 10^6^ cells in 1 ml of PBS were added to Falcon™ polystyrene round-bottom tubes (BD Biosciences) and stained with 1 μl of LIVE/DEAD^®^ Dead Cell Stain (Molecular Probes, Inc.) for 30 min on ice. The cells were washed twice with staining buffer and resuspended in 50 μl of staining buffer. To prevent nonspecific binding, the cells were incubated with 1 μg of 2.4G2 antibody specific for Fcγ III/II receptor (BioLegend, San Diego, CA) at 4°C for 5 min. Cells were then incubated with 0.5 μg of fluorophore conjugated rat anti-mouse CD3ε (BioLegend), rat anti-mouse CD4 (BioLegend), or isotype control for 30 min on ice, washed twice with staining buffer and resuspended in PBS with 2% (w/v) paraformaldehyde (Sigma) on ice for 15 min. To assess the percent of Treg cells, the cells were washed three times with PBS and resuspended in 1 ml PBS with 0.5% (v/v) Tween 20 (Sigma), washed three times with staining buffer and resuspended in 100 μl staining buffer containing 0.5 μg rat anti-mouse Foxp3 or isotype control (BioLegend) at room temperature for 30 min. The cells were washed three times with staining buffer, resuspended in 400 μl staining buffer, and analyzed immediately on the LSRII (BD Biosciences, San Jose, CA) and analyzed using FACSDiva software (BD Biosciences) and post-analyzed with FlowJo (Tree Star, Inc., Ashland, OR).

### 2.4 Statistics

Data were analyzed using GraphPad InStat® (GraphPad Software, Inc., La Jolla, CA). Skin graft rejection data were analyzed using the Log-rank (Mantel-Cox) test. Data for spleen weights and for flow cytometry results were analyzed by one-way ANOVA followed by Tukey’s multiple comparison test. Statistical significance was defined as *p* < 0.05.

## 3 Results

### 3.1 O-1966 Retards Skin Graft Rejection *In Vivo*

**FIGURE 1:**
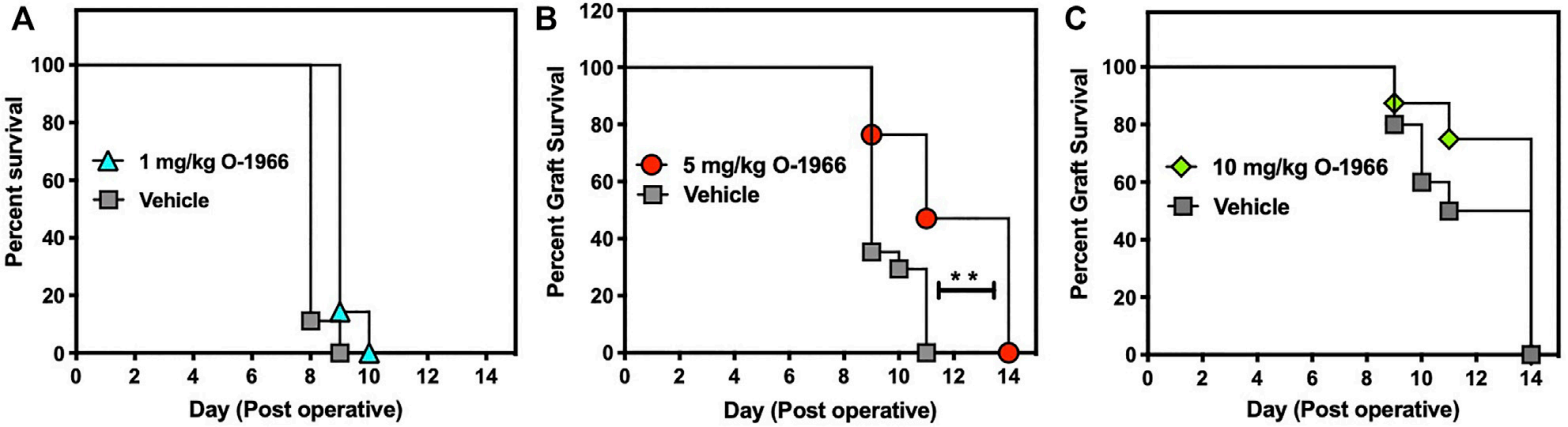
O-1966 treatment prolongs skin graft viability. Donor C3HeB/FeJ flank skin was transplanted to the back of a recipient C57BL/6J mice, sutured, and bandaged. Doses of O-1966 or vehicle (0.03% ethanol and 0.03% cremophor in saline) were injected i.p. every other day, from 1 h pre-op to 14 days post-op. On day 7 bandages were removed and grafts were monitored for rejection. Percent graft survival of mice treated with **(A)** 1 mg/kg O-1966 **(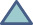)**, or vehicle **(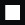)**, **(B)** 5 mg/kg O-1966 **(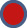)** or vehicle **(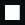)**, or **(C)** 10 mg/kg O-1966 **(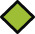)** or vehicle **(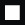)**. Panels **(A,C)** are results of a single experiment (n = 8 per group), and data in Panel **(B)** are the mean of two experiments (n = 17 per group). Median survival time of vehicle vs. O-1966 treatment at 5 mg/kg, **p < 0.001 by the Log rank [Mantel-Cox] test.

As shown in Figure 1, an inverse U-shaped dose response was observed for efficacy of O-1966 in retarding graft rejection. Mice that received either the 1 mg/kg or the 10 mg/kg doses showed no benefit from the CB2 agonist in graft prolongation (Figures 1A,C). In contrast, treatment with 5 mg/kg of O-1966 increased the median survival time of the grafts to 11 days compared to a median survival time of 9 days for vehicle-treated mice (*p* = 0.0004) (Figure 1B). The final rejection time for all grafts was extended from 11 days in controls to 14 days in the cannabinoid-treated animals.

### 3.2 O-1966 Treatment Decreases Splenic Weight in Skin Graft Recipients

**FIGURE 2:**
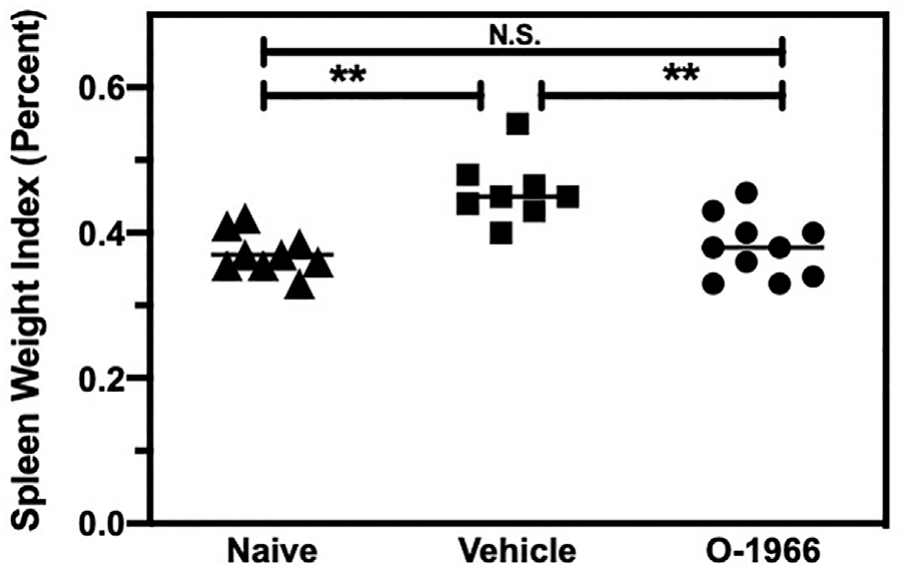
O-1966 decreases spleen weights in skin graft recipient mice. Donor C3HeB/FeJ flank skin was transplanted to the back of a recipient C57BL/6J mice, sutured, and bandaged. Doses of O-1966 (5 mg/kg) or vehicle (0.03% ethanol and 0.03% cremophor in saline) were injected i.p. every other day from 1 h pre-op to 14 days post-op. On post-op day 14, animals were sacrificed and spleens were removed. The spleen weight to body weight ratio was calculated and is expressed as a percentage, of control mice (▲) (n = 9) and grafted mice treated with 5 mg/kg O-1966 (■) (n = 8) or vehicle (●) (n = 10). **p < 0.001 for O-1966 vs. vehicle as determined by one-way ANOVA followed by Tukey’s multiple comparison test.

Mice that are mounting a significant allograft rejection response will show increased splenic weight due to the proliferation of responding T cells. Splenic weight was therefore determined in mice that had received skin grafts, with or without treatment with O-1966. On day 14 post-surgery, the spleens of graft recipient mice which had received the 5 mg/kg dose of O-1966 were removed, weighed, and normalized to their body weight to yield a splenic index. Figure 2 shows that the splenic indices of mice treated with O-1966 were significantly decreased compared to those of vehicle-treated mice. The splenic index of O-1966-treated mice was not different from that of control mice that did not receive skin grafts. Thus, the cannabinoid prevented the splenomegaly that characterizes animals undergoing skin graft rejection.

### 3.3 O-1966 Treatment Increases Treg Cells in Skin Graft Recipients

**FIGURE 3:**
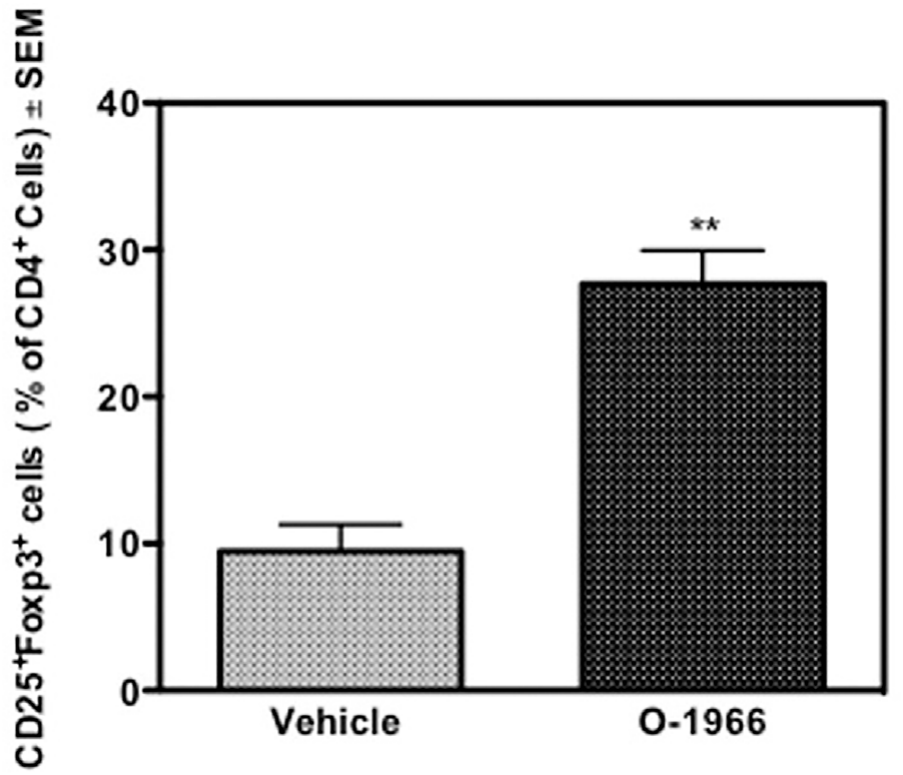
O-1966 treatment increases percentage of splenic Tregs in skin graft recipient mice. Donor C3HeB/FeJ flank skin was transplanted to the back of recipient C57BL/6J mice, sutured, and bandaged. Doses of O-1966 (5 mg/kg) or vehicle (0.03% ethanol and 0.03% cremophor in saline) were injected i.p. every other day from 1 h pre-op to 14 days post-op. Splenocytes were harvested from grafted mice treated with O-1966 **(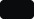)** or vehicle **(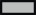)** on day 14 and were analyzed by flow cytometry for CD4^+^CD25^+^FoxP3^+^ Tregs (n = 17 for both groups). Data show number of Tregs as a percentage of total live CD4^+^ cells (LIVE/DEAD^®^ dead cell stain negative). Data are mean of two separate experiments. **p < 0.01 for O-1966 vs. vehicle as determined by one-way ANOVA followed by Tukey’s multiple comparison test.

**FIGURE 4:**
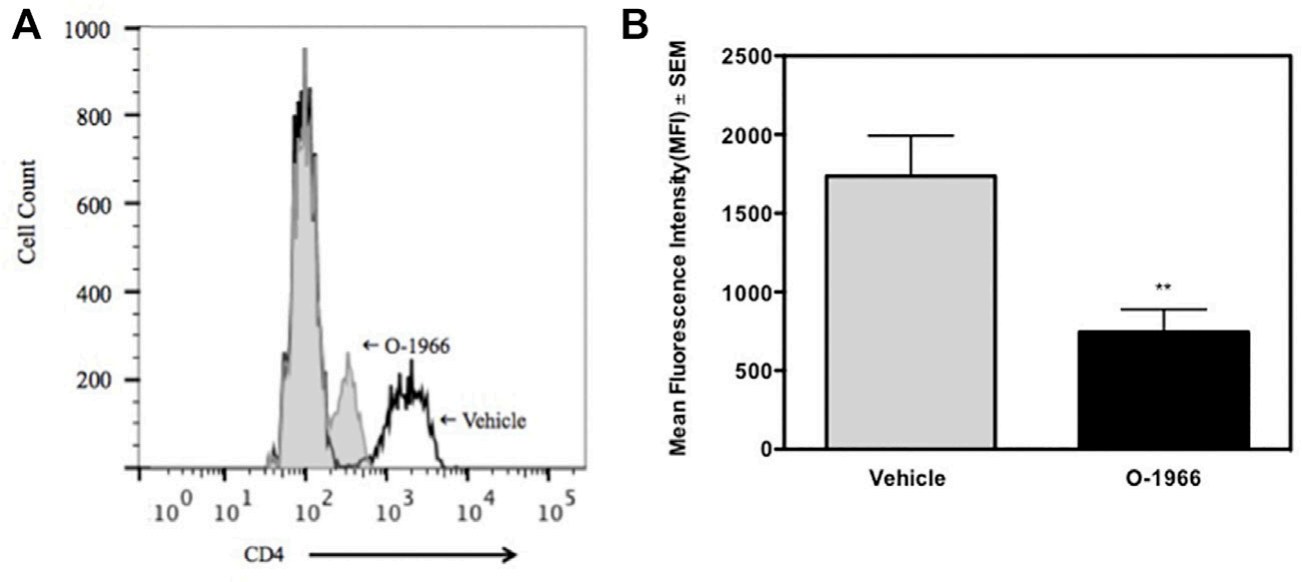
O-1966 treatment decreases CD4 expression in skin graft recipient mice. Donor C3HeB/FeJ flank skin was transplanted to the back of recipient C57BL/6J mice, sutured, and bandaged. Doses of O-1966 (5 mg/kg) or vehicle (0.03% ethanol and 0.03% cremophor in saline) were injected i.p. every other day from 1 h pre-op to 14 days post-op. Splenocytes were harvested on day 14, stained for CD4, and analyzed by flow cytometry. **(A)** Representative histograms of CD4 expression on CD3^+^ cells from mice treated with O-1966 (gray filled) or vehicle (white filled). **(B)** Mean fluorescence intensity (MFI) of CD4 in CD3^+^CD4^+^ populations from mice treated with O-1966 **(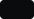)** or vehicle **(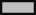)**. Data are mean of two experiments (n = 17 for both groups). **p < 0.01 for O-1966 vs. vehicle by one-way ANOVA followed by Tukey’s multiple comparison test).

The spleens of the animals that were sacrificed 14 days after surgery and weighed, were further processed to determine the percentage of Treg cells and the levels of CD4 expression on T cells. Figure 3 shows that mice treated with O-1966 had 27.7% CD25^+^Foxp3^+^ Tregs in the live CD4^+^ population, while mice treated with vehicle had only 9.5% CD25^+^Foxp3^+^ Tregs. This result leads to the conclusion that the CB2 selective agonist, O-1966, had the effect of increasing splenic Treg cells in mice that had received skin grafts. It was also found that mice treated with O-1966 had reduced levels of CD4 on the cell surface of CD3^+^ cells. O-1966 treatment caused a negative shift of fluorescence intensity of these cells. Figures 4A,B present the mean fluorescence intensity of CD4 from a representative animal and from all recipient mice (n = 17 for each treatment group), respectively, sand show that the average intensity of CD4 expression on the cell surface is decreased by O-1966 treatment.

### 3.4 *In Vivo* O-1966 Treatment Suppresses Splenocyte Proliferation *Ex Vivo* in the Mixed Lymphocyte Reaction

**FIGURE 5:**
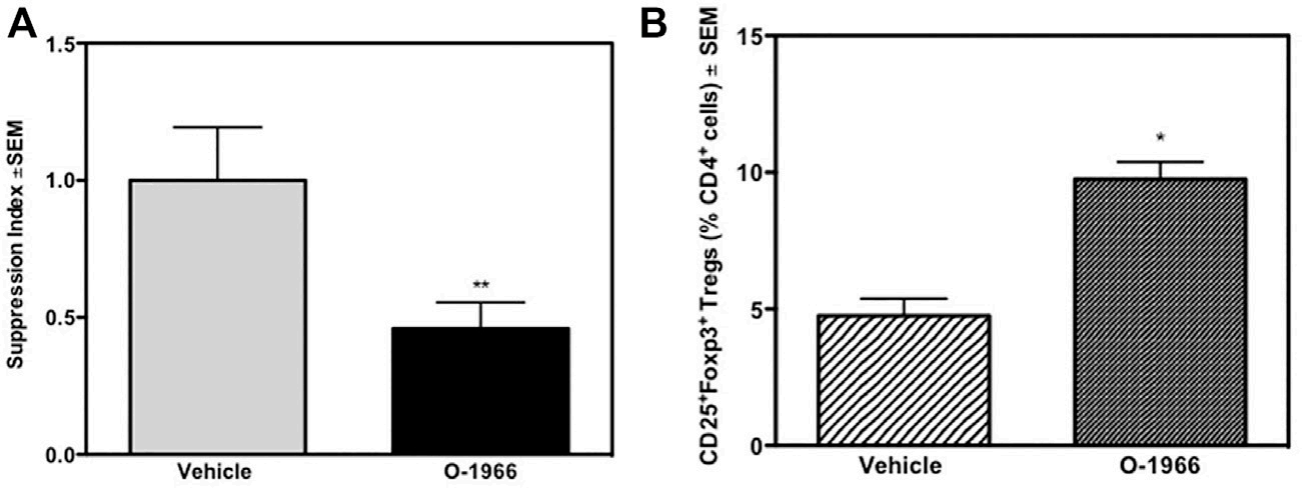
*In vivo* O-1966 treatment decreases proliferation and increases the percentage of Tregs following *ex vivo* stimulation. Donor C3HeB/FeJ flank skin was transplanted to the back of recipient C57BL/6J mice, sutured, and bandaged. Doses of O-1966 (5 mg/kg) or vehicle (0.03% ethanol and 0.03% cremophor in saline) were injected i.p. every other day from 1 h pre-op to 14 days post-op. On post-op day 14, animals were sacrificed and spleens were aseptically removed, restimulated with C3HeB/FeJ splenocytes and put into culture for MLR **(A)** or harvested at 48 h and analyzed by flow cytometry **(B)**. **(A)** Proliferation of cultures with splenocytes from O-1966 treated mice **(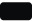)** or vehicle treated mice **(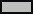)**. **(B)** Cultures harvested at 48 h from mice treated with O-1966 **(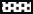)** or vehicle **(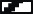)** and analyzed by flow cytometry for CD25^+^Foxp3^+^ Tregs (n = 17 for both groups). Data show number of Tregs as a percentage of total live CD4^+^ cells (LIVE/DEAD^®^ dead cell stain negative). Data are mean of two separate experiments. *p < 0.05, **p < 0.01 for O-1966 vs. vehicle by one-way ANOVA followed by Tukey’s multiple comparison test).

The responsiveness of splenocytes from C57BL/6J mice that had received an allograft 14 days prior, and were treated over the 14-day period with either O-1966 or vehicle, were harvested and placed in culture. These C57BL/6J cells were then restimulated *ex vivo* in an MLR assay with mitomycin-treated C3HeB/FeJ spleen cells, the same haplotype as the tissue that was grafted *in vivo*. As shown in Figure 5, splenocytes from mice grafted with C3HeB/FeJ skin and treated with O-1966 *in vivo* had significantly decreased proliferation in response to *ex vivo* stimulation with the C3HeB/FeJ cells.

### 3.5 *In Vivo* O-1966 Treatment Increases Treg Cells in an *Ex Vivo* Mixed Lymphocyte Reaction

**FIGURE 6:**
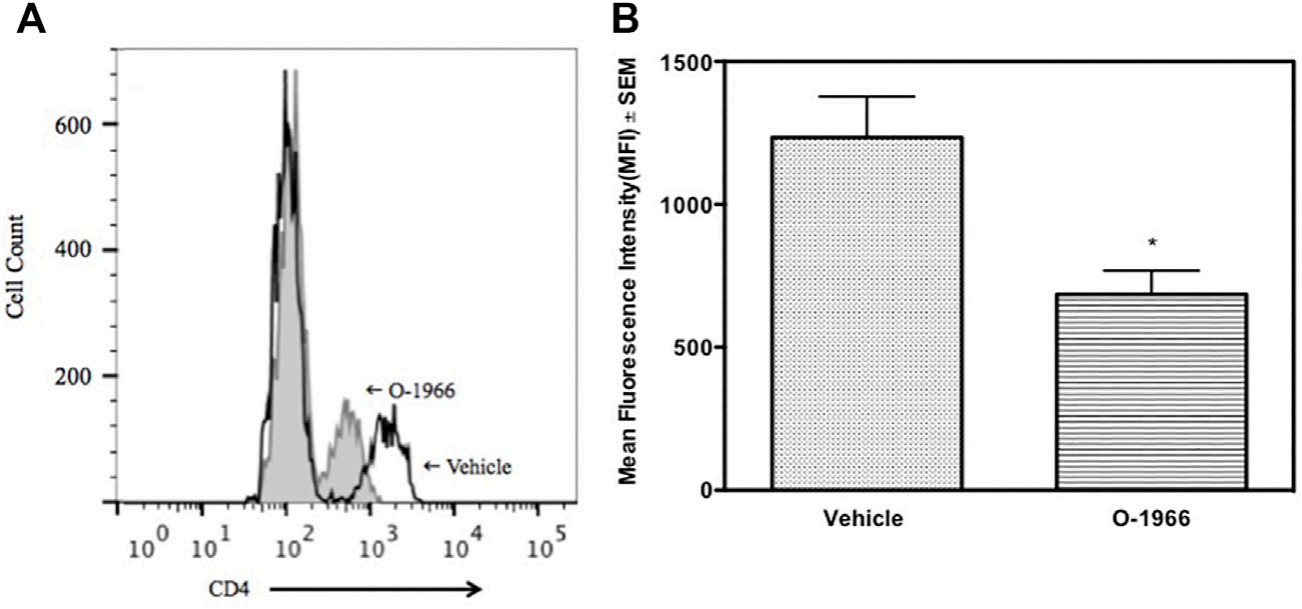
*In vivo* O-1966 treatment decreases CD4 expression following *ex vivo* stimulation. Donor C3HeB/FeJ flank skin was transplanted to the back of recipient C57BL/6J mice, sutured, and bandaged. Doses of O-1966 (5 mg/kg) or vehicle (0.03% ethanol and 0.03% cremophor in saline) were injected i.p. every other day from 1 h pre-op to 14 days post-op. On post-op day 14, animals were sacrificed and spleens were aseptically removed, restimulated with C3HeB/FeJ splenocytes and harvested at 48 h and analyzed by flow cytometry for CD4 expression. **(A)** Representative histogram of CD4 expression on CD3^+^ cells in cultures of splenocytes from skin graft recipient mice treated with O-1966 (gray filled) or vehicle (white filled). **(B)** Mean fluorescence intensity (MFI) of CD4 in CD3^+^ populations in cultures from O-1966 treated mice **(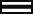)** or vehicle treated mice **(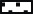)**. Data are representative of two experiments **(A)** or mean of two separate experiments **(B)**. *p < 0.05 for O-1966 vs. vehicle by one-way ANOVA followed by Tukey’s multiple comparison test).

Some of the wells of the *ex vivo* MLR cultures were harvested 48 h after the start of the assay and stained for CD4, CD25 and Foxp3, and analyzed by flow cytometry. Figure 6A shows that cultures from mice treated with O-1966 *in vivo*, and restimulated *ex vivo* had double the percentage of CD25^+^Foxp3^+^ Tregs compared to cultures using spleen cells taken from vehicle treated mice, with the percentage increasing from 4.7 to 9.8%. Further, *ex vivo* restimulated cells harvested from cannabinoid-treated mice had reduced levels of CD4 compared to cells from mice treated *in vivo* with vehicle (Figure 6B).

## 4 Discussion

The results of this study extend published *in vitro* studies using the MLR assay (Robinson et al., 2013; Robinson et al., 2015) to show efficacy of a CB2 selective agonist, O-1966, *in vivo*, in retarding rejection of skin grafts in mice and production of an immunosuppressive phenotype in the spleens of grafted animals injected with the cannabinoid. Spleens of grafted mice treated with O-1966 had significantly increased numbers of immunosuppressive Treg cells, which would be expected to dampen immune responses to the graft. The smaller spleen sizes of the grafted, treated animals, also indicates that CB2 administration led to inhibition of immune cell proliferation to the graft. Other investigators have reported that other CB2 selective agonists can reduce spleen weight (Gu et al., 2017). The current observation that spleen cells harvested from grafted, CB2-treated mice were inhibited in their proliferation when placed *ex vivo* in culture with cells of the mouse strain that supplied the graft, is powerful evidence that CB2 can mediate immunosuppression. A mechanism was identified for the immunosuppression, namely the induction of Treg cells. A CB2 receptor agonist has also been shown to induce Treg cells and IL-10 in a murine model of Crohn’s disease (Leinwand et al., 2017). Other possible mechanisms for CB2-mediated immunosuppression are suggested by studies from other laboratories and include blockage of T cell receptor signaling (Börner et al., 2009), and inhibition of maturation of T cells *in vivo* (Ziring et al., 2006). Another relevant paper reported that absence of the CB2 receptor resulted in more severe graft-versus-host reactions in a murine model by increasing CD8 cytotoxic T cells (Yuan et al., 2021).

In addition, it has been reported that JWH133, another CB2 selective agonist, protected against a murine model of ulcerative colitis by inducing T cell apoptosis (Rieder et al., 2010; Singh et al., 2012). In our previous *in vitro* studies using the MLR assay, we tested extensively for apoptosis and did not find it using O-1966 or another CB2 selective agonist, JWH-015 (Robinson et al., 2013). Consonant with our findings, lack of a cytotoxic effect on human, primary T cells by anandamide, an endogenous cannabinoid agonist, has been reported (Cencioni et al., 2010). Anandamide and JWH-015 (a CB2 selective agonist) were found to suppress activated T cells from producing IL-2, TNF-α and IFN-γ via action through the CB2 receptor (Cencioni et al., 2010). We had previously reported that O-1966 blocked IL-2 production in the MLR *in vitro* (Robinson et al., 2013). The experiments in this paper have focused on the effects of a CB2 selective agonist on T cells. However, the immunosuppressive capacity of CB2 agonists *in vivo* may reflect actions on macrophages. Several investigators have shown that CB2 agonists can polarize macrophages from an M1 to an M2 anti-inflammatory phenotype, where they produce increased amounts of IL-10 and arginase, and decreased amounts of pro-inflammatory cytokines and chemokines (Braun et al., 2018; Wu et al., 2020). In a mouse model of multiple sclerosis, treatment with a CB2 agonist markedly reduced microglial activation and reduced myeloid progenitor cell recruitment, possibly through altering the pattern of chemokine expression (Palazuelos et al., 2008). In contrast to these results, it has been reported that Δ^9^-THC attenuated skin graft rejection in mice via a CB1 mediated induction of myeloid-derived suppressor cells (Sido et al., 2015). Since Δ^9^-THC, like anandamide, binds to both CB1 and CB2 receptors, differentiating the receptor mediating the biological effect requires use of selective antagonists and cannabinoid receptor knock-out mice. The reason for the discrepancies in the literature are not readily apparent. CB2 agonists have also been shown to reduce the infarct size in induced stroke (Zhang et al., 2007) by preventing leukocyte extravasation at the site of the injury (Zhang et al., 2009). The effect has been narrowed to show that a CB2 agonist can inhibit neutrophil recruitment to the brain (Murikinati et al., 2010) and decrease the permeability of blood-brain barrier (Ramirez et al., 2012). Another group has also reported that CB2 receptor knock-out mice have defective neutrophil recruitment (Kapellos et al., 2019). A CB2 agonist has been shown to ameliorate sickness behavior induced by bacterial lipopolysaccharide that is mediated by excess production of pro-inflammatory cytokines (Sahu et al., 2019). There are several reports of CB2 attenuating sepsis in animal models (He et al., 2019). The CB2 receptor has also been reported to be protective against the inflammatory sequelae of several infections including HIV and SARS-CoV2 (Rizzo et al., 2020; Rastegar et al., 2021) and to protect against liver damage due to Concanavalin A in mice, which is a model for hepatotoxicity induced by Hepatitis B infection (Huang et al., 2019). Thus, CB2 agonists may target many different cells in the immune system to reduce inflammation, innate immunity and adaptive immunity. Although the immunosuppressive effect of O-1996 in this study is attributed to increased numbers of Treg cells, it would be appropriate to study whether this cannabinoid and other CB2 selective agonists engage other mechanisms to suppress the immune response.

In regard to the experimental design, experiments with cannabinoids have frequently been subject to criticism about the doses of drugs needed to induce biological effects. O-1966 has an affinity for the CB2 receptor of 23 nM (Wiley et al., 2002). Yet in the present experiments, an inverted U-shaped dose response curve was observed, with enhanced graft survival observed at the 5 mg/kg dose but not at 1 or 10 mg/kg. These higher doses, which do not seem to correlate with affinity constants, are in the range reported by other investigators who have used cannabinoids with activity at the CB2 receptor (Adhikary et al., 2011; Gu et al., 2017).

The potential use of CB2 selective agonists to retard graft rejection is attractive. The increase in mean graft survival time by 2 days is comparable to that achieved in mice using calcineurin inhibitors (Lagodzinski et al., 1990). Current immunosuppressive therapies (calcineurin inhibitors) used to prevent or block tissue rejection in organ transplantation are associated with significant untoward effects. For example, toxicity with chronic tacrolimus use is associated with post-transplantation diabetes mellitus (PTDM) due to the death of pancreatic islet cells (Taylor et al., 2005), and also with kidney damage. Tacrolimus and rapamycin use may induce hypertension linked to hyperkalemia (Hoorn et al., 2011). Up to 50% of transplant patients have renal dysfunction within 5 years of starting immunosuppressive therapy (Naesens et al., 2009; Hoskova et al., 2017). In addition, rapamycin may cause encephalopathies and other central nervous systems deficits including tremors, headache, convulsions and psychosis (Ho et al., 1996). It may be possible to reduce the doses of these standard therapies used to inhibit rejection by combining them in a reduced dose with a CB2 selective agonist like O-1966.

The synthetic cannabinoid studied in this manuscript. O-1966, is a CB2 selective agonist. As CB2 receptors are only sparsely expressed in the neural system, and their main expression is on cells of the immune system, psychoactive effects are not present. Rather, this class of synthetic cannabinoids, CB2 selective agonists, has the potential to be therapeutic agents for conditions where the immune system is over-active, such as graft rejection and autoimmune diseases. The current experiments add to the literature supporting use of this class of compounds to dampen immune responses.

## Data Availability

The datasets presented in this article are not readily available because the original contributions presented in the study are included in the article, further inquiries can be directed to the corresponding author. Requests to access the datasets should be directed to TE, tke@temple.edu.
